# Randomized trial of the efficacy of trial-based cognitive therapy for obsessive-compulsive disorder: preliminary findings

**DOI:** 10.47626/2237-6089-2021-0247

**Published:** 2023-08-04

**Authors:** Eleonardo Pereira Rodrigues, Ana Jardel Batista Fechine, Antonio César Oliveira, Cristiane Francisca Ferreira Matos, Cristiane de Medeiros Passarela, Curt Hemanny, Francimeuda de Morais Dias, José Wilson Batista, Luciana de Carvalho Nogueira Albuquerque, Myrla Sirqueira Soares, Pedro Evangelista Coelho, Vanessa Pires de Carvalho Araújo, Zila Mendes Ayres, Irismar Reis de Oliveira

**Affiliations:** 1 Universidade Federal da Bahia Salvador BA Brazil Programa de Pós-Graduação em Medicina e Saúde, Universidade Federal da Bahia, Salvador, BA, Brazil.; 2 Laboratório de Neurociência Cognitiva Universidade Estadual do Piauí Teresina PI Brazil Laboratório de Neurociência Cognitiva, Curso de Psicologia, Universidade Estadual do Piauí, Teresina, PI, Brazil.; 3 NYC Cognitive Therapy New York NY USA NYC Cognitive Therapy, New York, NY, USA.; 4 Universidade Federal da Bahia Salvador BA Brazil Programa de Pós-Graduação em Processos Interativos dos Órgãos e Sistemas, Universidade Federal da Bahia, Salvador, BA, Brazil.

**Keywords:** Obsessive-compulsive disorder, cognitive-behavioral therapy, trial-based cognitive therapy, exposure and response prevention, randomized clinical trial

## Abstract

**Introduction:**

Obsessive-compulsive disorder (OCD) is the fourth most prevalent mental disorder and is a disabling condition. OCD is associated with anatomical and functional changes in the brain, in addition to dysfunctional cognitions. The treatments of choice are selective serotonin reuptake inhibitors, cognitive-behavioral therapy (CBT), and exposure and response prevention (ERP). Trial-based cognitive therapy (TBCT) is a recent and empirically validated psychotherapy with a focus on restructuring dysfunctional negative core beliefs (CBs). The objective of this study was to evaluate the efficacy of TBCT relative to ERP for treatment of OCD.

**Methods:**

A randomized, single-blind clinical trial was conducted, randomizing 26 patients for individual treatment with TBCT (n = 12) or ERP (n = 14). The groups were evaluated at baseline, at the end of 3 months (12 sessions), and at 3, 6, and 12-month follow-ups.

**Results:**

Both approaches reduced the severity of symptoms with large effect sizes. These results were maintained at the 12-month follow-up assessment.

**Conclusion:**

TBCT may be a valid and promising treatment for this disorder.

## Introduction

Obsessive-compulsive disorder (OCD) is characterized by occurrence of obsessions, which are intrusive, repetitive, undesirable, inappropriate thoughts, and compulsions, which are characterized by behaviors that the individual performs in a ritualistic and repetitive way in response to obsessions.^1^ Approximately 60-70% of OCD patients experience sensory phenomena consisting of aversive or uncomfortable sensations or perceptions that drive repetitive behaviors (e.g., physical tension associated with anxiety).^2^ OCD is the fourth most frequent mental disorder, affecting males and females in equal proportions, and with prevalence of 1 to 3% of the world population.^3 - 6^ It is one of the most disabling conditions and it is among the ten diseases with the most significant functional impairment,^7 , 8^ with half of those affected presenting suicidal thoughts.^1^ The disorder is associated with extensive disability that affects all aspects of functioning, increased healthcare use, and diminished quality of life.^9^

The treatments of choice for OCD are selective serotonin reuptake inhibitors (SSRIs) combined with exposure and response prevention (ERP) or cognitive-behavioral therapy (CBT).^10 , 11^ From a psychotherapeutic point of view, ERP is considered the gold standard treatment for the disorder.^12 - 14^

ERP consists of directly exposing patients to stimuli that evoke obsessions and sensory phenomena while preventing them from executing compulsions.^11^ Although ERP is more effective than placebo and at least equal to the first choice SSRI drugs for treatment of OCD,^15 , 16^ the proportion of OCD participants who respond to treatment is around 55%.^17 , 18^ Findings show that there is low adherence to the treatment, because of the high level of anxiety produced by exposure.^11 , 14^

Cognitive therapy (CT) was proposed by Aaron Beck,^19^ in the early 1960s, as a structured psychotherapy model whose focus is modification of dysfunctional thoughts and beliefs. Several models have emerged since its initial development, resulting in cognitive-behavioral therapies (CBTs), a label given to a diverse range of psychotherapies that, despite philosophical differences, emphasize emotional, cognitive, and behavioral changes.^20 - 22^ Early studies comparing CT and ERP yielded comparable results.^23 - 25^

Trial-based cognitive therapy (TBCT) is one of the most recent approaches under the CBT umbrella,^26 , 27^ inspired by Franz Kafka’s novel, “The Trial.” The main TBCT techniques are analogous to a legal trial, in which the patient plays the roles of the defendant, the defense attorney, the prosecutor, the juror, the witnesses, and the judge.^28^ Although TBCT is a new CBT model, it has distinctive features such as a systematic set of step-by-step cognitive and behavioral techniques that integrate conventional CBT techniques; a new, organized, and systematic approach to modifying dysfunctional core beliefs (CBs) that simulates a court trial; an easy-to-remember and straightforward case formulation model that is easier for the patient to understand and for the therapist to use; and an integrative approach that allows cognitive, emotional, and experiential work to be done simultaneously.^29 , 30^ This approach acts on all three levels of cognition (automatic thoughts, underlying assumptions, and CBs) in three distinct phases, and, in the final stages, it focuses on development of metacognitive awareness, which is the ability to think critically about one’s cognitive functioning.^21 , 29^

TBCT is an empirically validated therapy, with evidence of efficacy for treatment of social anxiety disorder (SAD),^30 - 32^ post-traumatic stress disorder (PTSD),^33^ major depressive disorder (MDD),^21^ and possibly other disorders.^34^ Moreover, it has also been used as a preventive approach for adolescents in schools.^35^

The trial-based thought record (TBTR), a central TBCT technique, resulted in a better outcome than Greenberger and Padesky’s^36^ seven-column thought record used in combination with the positive data log in patients with SAD.^37^ In that study, rather than facilitating exposure to feared social situations, the purpose of both interventions was to modify CBs associated with SAD. Significant reductions in symptoms of social anxiety as well as physiological manifestations of anxiety were observed with both approaches, but participants receiving TBTR reported significantly greater reductions in fear of negative evaluation and social avoidance and distress, as well as greater improvements in quality of life.^32^

The TBCT protocol^27^ was used to evaluate TBCT efficacy for generalized SAD compared to a waitlist condition in a population with high rates of comorbid depression.^30^ Reductions in social anxiety, social avoidance, and depression were observed in the TBCT arm, all associated with large effect sizes, whereas, no differences between pre-treatment and post-treatment scores were observed in the waitlist condition. Interestingly, results also showed that comorbidity significantly moderated treatment efficacy, patients with comorbid conditions showing greater reductions in social anxiety symptoms across treatment relative to those with SAD only.

The efficacy of TBCT was compared to prolonged exposure (PE) in patients with PTSD in a randomized clinical trial (RCT) that recruited patients who met Diagnostic and Statistical Manual of Mental Disorders, 4th edition, Text Revision (DSM-IV-TR), criteria for PTSD.^33^ Patients were randomly assigned to receive either TBCT (n = 44) or PE (n = 51). A significant reduction in PTSD symptoms was observed in both TBCT and PE arms, but no significant difference between treatments was found. However, significant differences in depressive symptoms were observed, favoring TBCT, and the dropout rate was lower in the TBCT group relative to the PE group, suggesting that TBCT may be an effective alternative for treating PTSD.

An RCT also compared TBCT efficacy with the efficacy for MDD treatment of behavioral activation (BA) and treatment as usual (TAU).^21^ In this study, patients with MDD were randomized to one of three groups evaluated at baseline, after 6 weeks, and at week 12 (final evaluation). Both TBCT and BA (which also included antidepressants) were significantly better at reducing depressive symptoms than TAU (which involved antidepressants alone). TBCT and BA were also better than TAU at reducing disability and WHOQoL physical domain quality of life scores.^38^ The dropout rate was higher in the TAU group than in the TBCT and BA groups.

We identified six studies that compared CBT with ERP.^23 , 24 , 39 - 42^ As TBCT is a different format of CBT that modifies how CBT techniques are used and emphasizes CB change, we thought that, like CBT, TBCT could be at least as effective as ERP and should be tested as an OCD treatment. So, bearing in mind that (1) ERP for OCD has a high refusal and dropout rate^17^ ; (2) work in the area of obsessive-compulsive-relevant beliefs (e.g., inflated responsibility, thought-action-fusion, intolerance of uncertainty) suggests that a more beliefs-based intervention could be useful for individuals with OCD^11^ ; and (3) TBCT was characterized as a transdiagnostic approach, effective in the treatment of SAD,^31^ MDD,^21^ and PTSD^33^ ; we decided to compare the efficacy of TBCT relative to ERP in the treatment of OCD.

## Methods

### Design and participants

Seventy-five patients were assessed for participation, but only 26 who were diagnosed with OCD according to the DSM-IV criteria^43^ and scored ≥ 16 on the Yale-Brown Obsessive-Compulsive Scale (Y-BOCS)^44^ were enrolled. Only 22 of these patients completed the post-treatment (TBCT = 9 and ERP = 13). The study sample included 14 female (61.54%) and eight male (38.46%) adults, aged from 18 to 60 years (mean [standard deviation {SD}] = 32.8 [11.3]). All patients were using antidepressant medications for at least 3 months at a stable dose before inclusion in the study; they were requested to maintain their dosages unchanged during the active treatment. Patients who were undergoing psychotherapy or had had previous treatment with TBCT or ERP, and patients with neurological or mental disorders (mental retardation, psychotic disorder, severe personality disorder, or report of ongoing substance abuse disorders) that could compromise understanding and completion of the scales were excluded. All patients who completed the treatment were assessed for follow-up.

With regard to comorbidity, nine (41%) of the patients had a comorbid Axis I disorder (DSM-IV criteria). Regarding OCD presentation, our sample comprised “mixed” OCD subjects, i.e., they had both obsessions and compulsions and scores ≥ 16 on the Y-BOCS severity scale, according to criteria proposed by Shetti et al.^45^

### Procedures

Data collection was carried out (at baseline, post-treatment, and follow-up) by trained and experienced interviewers who were blind to the intervention groups and were available to answer questions and provide explanations when necessary.

Patients were recruited through public health services, health professionals’ offices, and publicity. Volunteers who agreed to participate in this study read and signed an informed consent form, were evaluated for inclusion criteria, underwent collection of clinical and sociodemographic data, and were randomized by the research coordinator to the ERP or TBCT groups. The interventions consisted of 12 individual psychotherapy sessions, with a weekly 1-hour session, and were conducted from 2014 to 2017 in Teresina, Brazil, at the Biotechnology Research Center, Science and Health Center at the State University of Piauí (Centro de Pesquisa em Biotecnologia, Centro de Ciência e Saúde, Universidade Estadual do Piauí [UESPI]). During follow-up, both intervention groups were reevaluated at 3, 6, and 12 months after the end of the treatment. The present study only reports data from the 12-month follow-up.

The study follows the principles of the Declaration of Helsinki and was approved by the Institutional Review Board (IRB) at the UESPI Health Sciences Center (Centro de Ciências da Saúde) (CCS/UESPI; Opinion 880996, CAAE 16179313.1.0000.5209), and was conducted in accordance with Brazilian National Health Council Resolution 466/2012. This trial is registered on ClinicalTrials.gov (ID: NCT02656784).

### Instruments

Patients were evaluated using scales and inventories to obtain sociodemographic and clinical data. The primary outcome measure was the Y-BOCS,^44^ and secondary outcome measures were the Beck Depression Inventory (BDI)^46^ and the Beck Anxiety Inventory (BAI).^47^ Evaluations took place at baseline, after sessions 6 and 12, and at 3, 6, and 12-month follow-ups. The evaluators were blinded to the intervention group.

#### Diagnostic interview

The Structured Clinical Interview for DSM-IV (SCID-I/P),^48^ as validated for the Brazilian population,^49^ was used for diagnosing Axis I (depression, anxiety) and Axis II (mental retardation and personality disorder) psychiatric disorders.

#### OCD severity

The Brazilian Portuguese version of the clinician-rated Y-BOCS was used to assess OCD symptom severity. This scale has been translated and validated for the Brazilian population (Cronbach’s alpha = 0.89).^44 , 50^ The Y-BOCS is composed of items that assess obsessions and compulsions and has a maximum score of 40 points. Scores from 8 to 15 are considered mild; 16 to 23, moderate; 24 to 31, severe; and 32 to 40, extreme. Scores ≥ 16 indicate clinical OCD.^51^ Since we used the Y-BOCS to measure the severity of OCD symptoms, we decided to analyze clinically significant change obtained with TBCT and ERP.^52^

#### Secondary outcome measures

Depressive symptoms were assessed using the BDI,^46^ a self-administered scale with 21 items and a maximum score of 63 points (Cronbach’s alpha = 0.80).^53^ Anxiety symptoms were assessed using the BAI,^47^ an instrument consisting of 21 items, with a maximum score of 63 points (Cronbach’s alpha = 0.88-0.92).^54^ Both inventories have been translated and validated for the Brazilian population.^55^

## Therapists

Interventions were delivered by three psychologists specialized in behavioral therapy for the ERP group and by three psychologists specialized in CBT for the TBCT group. All professionals had at least 6 years of clinical practice and were experienced in OCD treatment. The sessions were audio-recorded and evaluated by two supervisors, CM and ER, for ERP and TBCT respectively, to ensure fidelity to the protocols. ERP therapists were trained in Foa and Kozac’s model^56^ by one of the authors (CM), who was also in charge of their weekly supervision during the study. The TBCT therapists were initially trained at a 3-day workshop conducted by another of the authors (IRO), who developed TBCT,^27^ and were supervised weekly throughout the study by the lead author (ER).

## Treatments

### Trial-Based Cognitive Therapy

The TBCT course is described over 12 sessions, according to its manual for clinicians.^27^ The protocol presented here is restricted to clinical trials. However, in day-to-day clinical care, the structure is flexible and can last much longer than 12 sessions. The TBCT approach is organized on three levels in three distinct phases, during which the therapist and patient engage in discovery, assessment, and restructuring of dysfunctional cognitions.^27 , 28^

The first phase corresponds to the beginning of the treatment and involves presentation of the cognitive model using the TBCT conceptual diagram and comprises the initial sessions. The second phase comprises sessions 5 to 9 and includes the Trial I technique, in which the patient plays the role of the different characters in a court room, and employs an appeal preparation homework assignment to enable the patient to become better prepared for further trials to restructure additional dysfunctional CBs, such as “I’m vulnerable, weak, defective, irresponsible, mean, different,” and so on. The last phase encompasses sessions 10 to 12, aiming to engage the patient in a process of metacognitive awareness consolidation, observing the nature of thoughts, and realizing that there is no need to behave according to them, since they are dysfunctional. This is the TBCT relapse prevention phase.

## Exposure and response prevention

The ERP treatment used was based on the Foa and Kozak protocol,^56^ adjusted to last 12 sessions. The behavioral model of OCD indicates that a stimulus elicits obsessive emotional responses of fear and anxiety (acquired by respondent conditioning), evoking operative responses (compulsions) attempting to remove or reduce anxiety and obsessions, thus resulting in negative reinforcement of compulsive responses. The mechanism of clinical change in ERP is habituation to emotional responses and elimination of the escape/avoidance behavior.^11 , 17^

The first sessions of the ERP protocol^56^ consist of explaining the behavioral model of OCD. A hierarchy of feared situations of lesser to higher intensity is established, starting in the second session and assessed using the Subjective Units of Distress Scale (SUDS).^57^ The patient is instructed not to perform compulsions during the exposure, even if he or she feels compelled to perform them. *In-vivo* exposure is then applied, consisting of up to 1 hour of coping with the dreaded stimuli without responding with compulsions. Exposures to stimuli with low subjective discomfort that are started during the sessions are then assigned to be implemented as homework. From session 6 on, the patient is encouraged to be exposed to stimuli with SUDS scores between 6 and 7, on a scale ranging from 0 to 10. From session 9 to session 12, the patient is invited to be exposed to stimuli with scores higher than 7.^56 , 57^

## Statistical analyses

The software used for statistical analyses and data storage was the Statistical Package for Social Sciences - SPSS, version 22.0 (SPSS Inc., Chicago, USA). Descriptive statistical analyses were performed with data on prevalence, percentages, means, and SDs. In the descriptive analyses, the chi-square test (χ^2^ ) or Fisher’s exact test were used for dichotomous variables and mean differences between groups at baseline were assessed with Student’s *t* test and analysis of variance (ANOVA).

Missing data were analyzed by intention-to-treat (ITT), which included all patients randomized in the study, regardless of when they dropped out,^58^ and with multiple imputation (MI),^59^ which imputed five databases including age, sex, and intervention group, adopting the missing at random assumption (MAR). Generalized estimating equations (GEE) with a first-order autoregressive work matrix were used to compare the efficacy measures between TBCT and ERP groups and over time (initial and final evaluations). The time x group interaction was also analyzed. The level of statistical significance assumed was 0.05.

Cohen’s *d* effect sizes (ESs) were calculated between the groups, with values of 0.20, 0.50, and 0.80 being considered, respectively, small, medium, and large effect sizes.^60^ All outcome measures entered in the between-group analysis were computed. A positive *d* was in favor of ERP and a negative one in favor of TBCT. Although small, a *d* > 0.25 was considered as having a possible clinical significance.^24^

## Results

### Participants

After screening 75 subjects, a sample of 26 patients was randomized (TBCT = 12; ERP = 14). Four patients dropped out after randomization (TBCT = 3; ERP = 1) and 22 subjects completed all of the psychotherapy sessions (TBCT = 9; ERP = 13).

**Figure 1:**
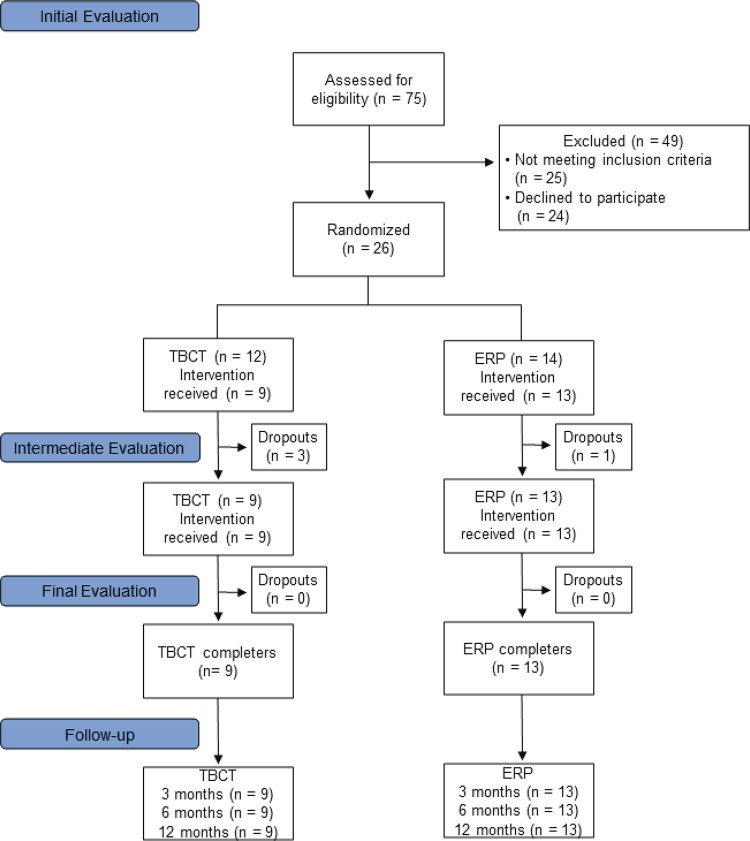
Illustrative flow diagram (Consolidated Standards of Reporting Trials [CONSORT]).61 ERP = exposure and response prevention; TBCT = trial-based cognitive therapy.

In this study, 61.54% of the sample were women (n = 16) and no differences were observed between groups regarding gender (p = 0.75) or baseline clinical data: Y-BOCS: (F [1.24] = 0.500, p = 0.48); BAI: (F [1.24] = 0.001, p = 0.98); BDI: (F [1.24] = 0.766, p = 0.39). The mean age of the sample was 32.8 (SD = 11.3) years (TBCT = 32.4 [SD = 13.9] and ERP = 33.0 [SD = 11.1]). Figure 1 shows the study design flowchart.

### Main results

Differences within and between groups in primary measures

Table 1 shows the patients’ baseline and final Y-BOCS (total, obsessions, and compulsions), BDI, and BAI scores, with ITT and MI.

**Table 1:** Assessment of between- and within-group change in symptoms in an ITT sample using GEE

Measures	Baseline	Post-treatment	p* between-groups	p^†^ within-groups
	
	Mean (SD)	Mean (SD)		
Y-BOCS (total)				
TBCT	24.92 (6.94)	13.19 (9.36)	0.45	0.0005
ERP	26.79 (6.03)	15.24 (5.68)	-	0.001
Y-BOCS (obsessions)				
TBCT	12.83 (4.16)	6.64 (4.89)	0.25	0.0005
ERP	14.00 (2.84)	8.69 (3.08)	-	0.0005
Y-BOCS (compulsions)				
TBCT	12.08 (3.59)	6.53 (4.74)	0.80	0.002
ERP	12.79 (3.63)	6.52 (3.08)	-	0.0005
BDI				
TBCT	25.17 (13.38)	8.89 (10.08)	0.66	0.0005
ERP	20.79 (11.30)	10.42 (9.07)	-	0.0005
BAI				
TBCT	29.42 (13.02)	9.63 (10.78)	0.44	0.0005
ERP	29.57 (12.90)	15.14 (9.61)	-	0.0005

BAI = Beck Anxiety Inventory; BDI = Beck Depression Inventory; ERP = exposure and response prevention; GEE = generalized estimation equations; ITT = intention-to-treat; SD = standard deviation; TBCT = trial-based cognitive therapy; Y-BOCS = Yale-Brown Scale for Assessment of Obsessive-Compulsive Symptoms (obsessions, compulsions, and total). * p-value for inter-group difference (ERP taken as reference); ^†^ p-value for pre-treatment to post-treatment difference.

#### Y-BOCS (total)

There were statistically significant reductions in total Y-BOCS scores over time in both the TBCT and the ERP groups (β = -11.678, p < 0.001). There was no statistical difference in the reduction in scores when comparing TBCT and ERP (β = -1.194, p = 0.45). A complete case analysis did not detect any statistical differences between groups (p > 0.50).

The within-group analyses detected significant effects in the TBCT (β = -11.737, p < 0.0005) and the ERP (β = -11.628, p < 0.001) groups. In other words, there were significant decreases in the Y-BOCS scores within both TBCT and ERP groups from pre-treatment to post-treatment.

In terms of the interaction, results showed that there was no effect of time × group on Y-BOCS total scores (β = -0.109, p = 0.97), indicating that the reduction in symptoms over time (from pre-treatment to post-treatment) was not different between the treatment groups.

#### Y-BOCS (obsessions)

There were statistically significant reductions in Y-BOCS scores (obsessions) over time in both intervention groups in the complete cases analysis (β = -5.890, p < 0.001). Likewise, the analysis with MI showed significant reductions in this score in both psychotherapy groups over time (β = -5.739, p < 0.001).

In the parameter estimate comparisons, there was a lack of evidence of difference between TBCT and ERP concerning Y-BOCS-obsessions: (β = -1.572, p = 0.25). The result of the within-group analyses showed significant effects in the TBCT (Y-BOCS [obsessions; β = -6.284, p < 0.0005]) and ERP groups (Y-BOCS [obsessions; β = -5.272, p < 0.0005]). In terms of the interaction, results showed that there was no effect of time × group (β = -0.811, p = 0.65).

#### Y-BOCS (compulsions)

There was a statistically significant reduction in compulsions over time: (β = -5.967, p < 0.0005), which also occurred in the complete case analysis (β = -6.258, p < 0.0005). There were no statistical differences in reduction of scores between TBCT and ERP in the MI analysis: (β = -0.330, p = 0.80). The results of within-group analyses revealed significant effects in both TBCT (Y-BOCS [compulsions; β = -5.556, p = 0.002]); and ERP groups (Y-BOCS [compulsions; β = -6.302, p < 0.0005]), indicating that there were significant decreases in Y-BOCS (compulsions) scores in both TBCT and ERP groups from pre-treatment to post-treatment. In terms of the interaction, results showed that there was no effect of time × group (β = 0.726, p = 0.69).

## Clinically significant changes

We replicated the analysis conducted by Belloch et al.,^52^ who used the criteria proposed by Jacobson and Truax.^39^ Patient improvement and recovery at post-treatment and at follow-up were calculated according to the following criteria: improvement = YBOCS ≤ 12 plus decrease in YBOCS pre-treatment to post-treatment of at least six points; recovery = YBOCS ≤ 7 plus decrease in YBOCS pre-treatment to post-treatment of at least six points.

At post-treatment, 55.6% (five out of nine) patients in the TBCT arm met the criteria for improvement, relative to 23.1% (three out of 13) in the ERP group (Fisher’s exact test: p = 0.18). In turn, 33.3% (three out of nine) patients in the TBCT arm met the criteria for recovery, compared to 7.7% (one out of 13) patients in the ERP group (Fisher’s exact test: p = 0.26).

Also replicating the study by Belloch et al.,^52^ and considering that the 3, 6, and 12-month follow-up results were similar, here we only report data from the last of these periods. At the 12-month follow-up, 66.7% (six out of nine) patients in the TBCT arm met the improvement criteria, relative to 23.1% (three out of 13) in the ERP group (Fisher’s exact test: p = 0.07). In turn, 55.6% (five out nine) of completers in the TBCT arm met criteria for recovery compared to only 7.7% (one out of 13) in the ERP group (Fisher’s exact test: p = 0.02).

## Differences within and between groups in secondary measures

In both TBCT and ERP, there was a significant reduction in BDI scores over time (β = -13.148, p < 0.001), but no evidence that the treatments were different from each other in reducing depressive symptoms (BDI [β = 1.540, p = 0.66]). Within-group analysis showed a decrease in BDI scores from pre-treatment to post-treatment in the TBCT (BDI [β = -13.095, p = 0.001]) and ERP groups (BDI [β = -13.192, p < 0.001]). In terms of the interaction, results showed that there was no effect of time × group (β = -5.682, p = 0.36).

There was a reduction in the anxiety scores measured by BAI over time in both treatments (β = -16.943, p < 0.001). There was no evidence of statistical difference between TBCT and ERP (BAI [β = -2.743, p = 0.44]). Within-group analysis showed a decrease of BAI scores from pre-treatment to post-treatment in the TBCT (BAI [β = -18.135, p < 0.001]) and ERP groups (BAI [β = -15.921, p < 0.001]). In terms of the interaction, results showed that there was no effect of time × group (β = -5.177, p = 0.45).

## Effect sizes

Table 2 shows intra-group and inter-group ESs (Cohen’s *d* ). All variables presented large or very large intra-group ESs, with *d* > 0.80, at both post-treatment and 12-month follow-up. Between-group ESs were clinically significant, favoring TBCT for BDI and BAI ( *d* > -0.50 at post-treatment), and for all measures at 12-month follow-up ( *d* range: -0.33 to -0.58).

**Table 2:** Intra-group and inter-group effect sizes at post-treatment and 12-month follow-up

	Baseline	Post-treatment	12-month follow-up	*d* intra-group*	*d* between-group^†^	*d* intra-group^‡^	*d* between-group^§^
Measures	Mean (SD)	Mean (SD)	Mean (SD)				
Y-BOCS (total)							
TBCT	24.92 (6.94)	13.19 (9.36)	12.01 (2.65)	1.42	-0.02	2.46	-0.33
ERP	26.79 (6.03)	15.24 (5.68)	16.14 (2.27)	1.97	-	2.35	-
Y-BOCS (obsessions)							
TBCT	12.83 (4.16)	6.64 (4.89)	5.81 (1.47)	1.36	-0.24	1.94	-0.46
ERP	14.00 (2.84)	8.69 (3.08)	8.66 (1.29)	1.78	-	1.48	-
Y-BOCS (compulsions)							
TBCT	12.08 (3.59)	6.53 (4.74)	6.23 (1.31)	1.31	0.19	2.16	-0.17
ERP	12.79 (3.63)	6.52 (3.08)	7.59 (1.12)	1.86	-	1.97	-
BDI							
TBCT	25.17 (13.38)	8.89 (10.08)	8.41 (2.75)	1.37	-0.46	1.73	-0.57
ERP	20.79 (11.30)	10.42 (9.07)	11.30 (2.23)	1.01	-	1.52	-
BAI							
TBCT	29.42 (13.02)	9.63 (10.78)	9.21 (3.08)	1.65	-0.40	2.46	-0.48
ERP	29.57 (12.90)	15.14 (9.61)	15.88 (2.52)	1.26	-	1.48	-

BAI = Beck Anxiety Inventory; BDI = Beck Depression Inventory; ERP = exposure and response prevention; SD = standard deviation; TBCT = trial-based cognitive therapy; Y-BOCS = Yale-Brown Scale for Assessment of Obsessive-Compulsive Symptoms (obsessions, compulsions, and total). * Intra-group Cohen’s *d* at post-treatment (scores at baseline taken as reference); ^†^ Between-group Cohen’s *d* at post-treatment (ERP taken as reference); ^‡^ Intra-group Cohen’s *d* at 12-month follow-up (scores at baseline taken as reference); ^§^ Between-group Cohen’s *d* at 12-month follow-up (ERP taken as reference).

## Discussion

The results of this study indicate that both ERP and TBCT were able to reduce baseline symptom scores measured by Y-BOCS. Although both interventions helped to reduce the OCD symptoms, there was a lack of evidence of statistical difference between groups. Regarding follow-up, both TBCT and ERP maintained the therapeutic gains for 12 months.

In the present study, TBCT reduced symptoms by 47.1% from pre-treatment to post-treatment and ERP reduced symptoms by 43.1%; TBCT also reduced symptoms by 52.6% from pre-treatment to follow-up and ERP reduced symptoms by 44%. Thus, mean Y-BOCS scores for both TBCT and ERP were in the ranges observed in previous studies in terms of changes from baseline.^24 , 40 , 41^

Whittal et al.^40^ compared CBT with ERP delivered in an individual format and showed that there were no significant difference in Y-BOCS scores between the interventions at post-treatment or at 3-month follow-up. Our results were similar, but we were able to show a longer sustained gain over 12 months of follow-up in both TBCT and ERP. Those authors suggested that CBT was as effective as ERP when delivered individually and that CBT might be considered the treatment of choice in cases where ERP is difficult to administer, as is the case with primary obsessions. As the results of our study suggest, TBCT is at least as effective as ERP and is especially focused on restructuring CBs. Thus, a larger RCT to test use of TBCT in OCD patients compared to ERP and conventional CBT should be encouraged.

A study of 16 weeks’ duration^24^ compared CBT with intensive ERP in OCD patients. The response rate was similar in both groups. However, BDI scores at post-treatment were significantly more improved by CBT. The authors concluded that CBT and ERP were equally effective for OCD, but that at post-test CBT had specific effects on depression that were stronger than those in the ERP group. In the present study, it was not possible to demonstrate any difference between TBCT and ERP in terms of the effect on depressive symptoms, probably due to the small sample size. However, although the PTSD study comparing TBCT and PE^33^ did not show any significant difference in PTSD symptoms between groups, significant differences were observed in depressive symptoms that favored TBCT and the dropout rate was also lower in the TBCT group. Considering that TBCT has been shown to be effective in MDD,^21^ this intervention might be considered in OCD patients with comorbid depression. TBCT is a transdiagnostic approach that has also been shown to be effective in SAD^27^ with comorbid depression and PTSD.^33^ It might therefore be promising in OCD patients with such comorbid conditions.

Akin to what was found by Belloch et al.^52^ regarding mean total Y-BOCS scores pre-treatment, patients in our study fell into the range of severe OCD (TBCT = 27.8 and ERP = 27.7 in this study; CBT = 26.40 and ERP = 24.69 in the Belloch et al. study). Both their study and ours had small sample sizes and 12-month patient follow-up. Our results were comparable, suggesting that both TBCT and ERP were at least equally effective for treatment of severe OCD symptoms. However, using the same cutoffs for improvement and recovery that Belloch et al.^52^ used, our data showed that there were significantly more recovered patients in the TBCT treatment group at 12-month follow-up.

CBT focuses on identifying and restructuring distorted thoughts and dysfunctional CBs. Thus, acting on obsessions and reducing their frequency and intensity is one possible underlying mechanism of improvement. Unfortunately, we did not measure CBs. However, three TBCT studies^26 , 34 , 37^ demonstrated that there were reductions in the degree to which clients were attached to dysfunctional negative CBs and in associated negative emotions after a single session using the trial‐based thought record (TBTR), which is a central TBCT technique. Also, one of these studies^34^ indicated that inclusion of the empty‐chair technique during TBTR might boost efficacy for reducing attachment to CBs and the intensity of the accompanying emotions compared to the conventional static TBTR, possibly because of the empty chair’s more experiential nature.

On the other hand, ERP does not directly act to modify the cognitive contents of obsessions, but proposes confronting them, preventing performance of rituals.^17 , 23 , 62^ So, when the patient is exposed to stimuli that produce obsessions and anxious responses, resisting them, habituation occurs. Also, since the patient is not allowed to perform the compulsions, the feared consequences do not occur, leading to elimination of the escape/avoidance response (i.e., the rituals).^63^ In the present study, it is suggested that OCD symptoms were reduced both by the changes in the content of thoughts and beliefs by TBCT, without direct exposure, and by the change in the way of relating to cognitions by not performing compulsions.

Finally, we consider that both automatic thoughts and CBs are related to the meaning (interpretation) to be restructured in OCD. Although the main TBCT techniques target CBs such as “I’m vulnerable, weak, defective, irresponsible, mean,” and so on, the underlying assumptions are also targeted with behavioral experiments, optimized with two other TBCT techniques, namely: 1) the color-coded symptom hierarchy (CCSH), which tends to make behavioral experiments more palatable; and 2) consensual role-play (CRP), which also incorporates the experiential empty chair approach, making defying the rituals less challenging. The goal of both CCSH and CRP is to change the underlying assumptions (e.g., “If I forget to turn off the stove, then a catastrophe will ensue”) that maintain the OCD coping strategies (rituals), but this is done primarily by helping patients to change behaviors.

This study has a significant limitation, which is the small sample size. Considering that the required sample size was 32 patients per arm, unfortunately our study included fewer patients than the ideal number. Studies with small samples may produce false-positive results or overestimate the magnitude of an association.^64^ Although this is not a problem that could invalidate the study, the small sample requires greater caution in its interpretation. Four other possible limitations were 1) not having measured sensory phenomena, considering their high frequency in OCD patients; 2) although participants were asked to keep their medications stable, this was not monitored; 3) lack of evaluation of satisfaction with the interventions; and 4) the absence of a detailed and quantitative method to measure fidelity to the protocols. However, to minimize the last of these problems, the supervisors listened to the session recordings and discussed them with the therapists at the weekly supervision meetings.

As for its strengths, this study used MI, a modern approach to dealing with missing data. Moreover, it was possible to collect follow-up data, which enabled maintenance of the gains over 12 months to be observed. Further clinical research with TBCT and ERP should be conducted with larger samples, so that a possible difference (or not) between TBCT over ERP can be more accurately measured, as suggested by the results observed in this study.

## Conclusion

In conclusion, TBCT was not different from ERP in reducing obsessive-compulsive symptoms in OCD patients. Both TBCT and ERP were able to maintain the therapeutic gains at 12-month follow-up. However, TBCT yielded significantly more recovered patients.

This preliminary study suggests that further studies are needed to investigate the long-term effects of TBCT for OCD.
